# Primary Bone Lymphoma: A Review of the Literature with Emphasis on Histopathology and Histogenesis

**DOI:** 10.3390/diseases11010042

**Published:** 2023-03-02

**Authors:** Theofilos Kanavos, Effrosyni Birbas, Alexandra Papoudou-Bai, Eleftheria Hatzimichael, Aikaterini Kitsouli, Georgia Karpathiou, Panagiotis Kanavaros

**Affiliations:** 1 Department of Anatomy-Histology-Embryology, Faculty of Medicine, School of Health Sciences, University of Ioannina, 45110 Ioannina, Greece; 2 Department of Pathology, Faculty of Medicine, School of Health Sciences, University of Ioannina, 45110 Ioannina, Greece; 3 Department of Hematology, Faculty of Medicine, School of Health Sciences, University of Ioannina, 45500 Ioannina, Greece; 4 Department of Pathology, University Hospital of Saint-Etienne, 42055 Saint-Etienne, France

**Keywords:** bone, lymphomas, B-cells, immunohistochemistry

## Abstract

Primary bone lymphoma (PBL) is a rare neoplasm of malignant lymphoid cells presenting with one or more bone lesions without nodal or other extranodal involvement. It accounts for approximately 1% of all lymphomas and 7% of malignant primary bone tumors. Diffuse large B-cell lymphoma (DLBCL), not otherwise specified (NOS) represents the predominant histological type and constitutes over 80% of all cases. PBL may occur at all ages with a typical diagnosis age of 45–60 years and a slight male predominance. Local bone pain, soft tissue edema, palpable mass and pathological fracture are the most common clinical features. Diagnosis of the disease, which is frequently delayed due to its non-specific clinical presentation, is based on the combination of clinical examination and imaging studies and confirmed by combined histopathological and immunohistochemical examination. PBL can develop in any part of the skeleton, although it occurs most commonly in the femur, humerus, tibia, spine and pelvis. The imaging appearance of PBL is highly variable and unspecific. In terms of the cell-of-origin, most cases of primary bone DLBCL (PB-DLBCL), NOS belong to the germinal center B-cell-like subtype and specifically originate from germinal center centrocytes. PB-DLBCL, NOS has been considered a distinct clinical entity based on its particular prognosis, histogenesis, gene expression and mutational profile and miRNA signature. PBL carries a favorable prognosis, especially when treated with combined chemoradiotherapy.

## 1. Introduction

Primary bone lymphoma (PBL) is currently defined as a neoplasm composed of malignant lymphoid cells that presents with one or more bone lesions without nodal involvement or other extranodal lesions, according to the 2020 World Health Organization (WHO) classification of soft tissue and bone tumors [1,2]. The majority of PBLs represent non-Hodgkin lymphoma (NHL) and diffuse large B-cell lymphoma (DLBCL), not otherwise specified (NOS) is by far the most common histological type [3]. PBL must be distinguished from the secondary bone involvement of systemic lymphomas, which occurs in 16–20% of lymphoma patients and carries a poor prognosis, whereas PBL is considered to have the most favorable prognosis of all malignant primary bone tumors [4,5,6]. Diagnosis of PBL, which is frequently delayed due to its non-specific clinical manifestations and equivocal radiographic findings, is based on the combination of clinical examination and imaging studies and confirmed by histopathological examination with immunohistochemical staining [7,8,9]. The present review summarizes the epidemiological, clinical, radiological and histological features, as well as the etiology, histogenesis, treatment and prognosis of PBL.

## 2. Epidemiology

PBL is a rare neoplastic disease and accounts for approximately 1% of all lymphomas, 3–7% of extranodal lymphomas and 7% of malignant primary bone tumors [4,10,11,12]. NHL constitutes the vast majority of PBLs with DLBCL, NOS, representing over 80% of all cases [10,13,14]. PBL of T-cell origin is extremely uncommon, but has a higher relative frequency in Japan and Taiwan compared to the West [15,16]. PBL may occur at all ages, with a typical diagnosis age of 45–60 years [10,15,17]. In addition, men are affected more than women, with a male/female ratio of 1.2–1.8 [3].

## 3. Clinical Findings

The clinical features of PBL are generally unspecific, frequently leading to a delay in diagnosis, and, thus, a high index of suspicion is required [7,18]. The most common symptom of PBL is local bone pain in the affected area, which is not relieved by rest and has been characterized as insidious, intermittent and progressively worsening [2,3,4,7,9,15,18,19,20,21,22,23]. Other less common manifestations include soft tissue edema, palpable mass, pathological fracture, restricted range of motion in the involved articulation and “B” symptoms, namely fever, night sweats and unintentional weight loss [2,3,4,8,11,18,19,22,23,24,25]. The latter are present in a minority of patients and are less frequent in PBL compared to systemic lymphomas [2,24]. Nonetheless, PBL should be considered in cases of fever of unknown origin, especially in the presence of bone pain [20]. Spinal cord compression and hypercalcemia due to osteolysis with related symptoms, such as lethargy, somnolence and constipation, are two major complications [2,9,22,23,24]. Cases of PBL involving the mandible or maxilla can manifest with toothache, loose teeth, gingiva edema and local numbness and are, therefore, often misdiagnosed as oral diseases [26,27]. Cases with a history of preceding trauma in the affected area have also been reported [18,28,29,30]. The average time between the onset of symptoms and the diagnosis is 8 months [2,7].

PBL can develop in any part of the skeleton, although is most common in the femur, humerus, tibia, spine and pelvis [2,3,4,7,8,9,10,17,20,21,22,24,31]. Other less common sites of occurrence include the skull, forearm, scapula, clavicle, patella, hands and feet [3,4,9,10,17,21,25]. Interestingly, younger age has been associated with an appendicular location of PBL, which could be explained by the active bone marrow present in the long bones of younger patients [24]. In the case of long bones, PBL most frequently occurs in the metaphysis [17].

## 4. Radiological Findings

Although PBL diagnosis is based on histopathological examination requiring bone biopsy, imaging studies are crucial for the initial depiction, biopsy guidance, lesion extent determination and staging, as well as restaging and treatment response monitoring [24]. In addition, if a bone biopsy reveals NHL, imaging can help to determine whether this lesion is either a PBL or a bone involvement of a lymphoma originating in an extraosseous site. The imaging appearance of PBL is highly variable and unspecific [3,15].

Plain X-ray examination is the initial diagnostic modality of choice in patients with suspected PBL [22]. However, radiographic findings are equivocal, frequently leading to a diagnosis delay [7]. After reviewing 20 published cases, Krishnan et al. [32] identified three radiographic patterns that PBL can manifest with: (i) the lytic-destructive pattern; this pattern concerns the majority of PBL cases and can be further divided into the permeative and moth-eaten pattern of destruction. A periosteal reaction has also been reported and may be either multilayered or broken, with the latter indicating a poorer prognosis. Additionally, X-ray tests can demonstrate cortical interruption, sequestra and soft tissue masses, which are findings suggestive of a more aggressive neoplasm. (ii) Blastic-sclerotic pattern; an osteosclerotic appearance of PBL is scarce. Cases of lesions with mixed lytic and sclerotic areas have also been reported. The ivory vertebra sign, referring to the diffuse and homogeneous increase in the radiopacity of a vertebral body, can also be present in PBL. However, it is more frequently observed in cases of spinal involvement in Hodgkin’s disease [24]. It is worth mentioning that sclerotic areas can develop in PBL cases following chemotherapy or radiotherapy [32]. (iii) Subtle or “near-normal” findings; in some PBL cases, conventional X-ray examination fails to depict any notable finding despite the symptoms. Nevertheless, such cases may show remarkable abnormalities in more sensitive modalities, such as MRI, and, thus, further radiological evaluation is required.

Computed tomography (CT) plays a significant role in the management of PBL since it can effectively depict soft tissue extension, bone marrow involvement and cortical disruption. It is the primary modality for radiologically guided biopsy, which is essential for the definite diagnosis of PBL [24]. In addition, CT can be used for the staging, restaging and follow-up of PBL [22]. CT scans as well as plain radiographs can reveal potential pathological fractures related to PBL [15,22].

Magnetic resonance imaging (MRI) is vital for the imaging investigation of PBL. It is the modality of choice for the early detection of PBL and the depiction of its soft tissue extension and bone marrow involvement, as it can accurately demonstrate cortical erosion. MRI can also be used to evaluate the outcome of treatment and therapy-related complications, such as epidural lipomatosis, insufficiency fractures and myeloid depletion. However, MRI presents low specificity in the restaging of PBL [24].

MRI findings of PBL can be “protean” and even resemble benign entities [24]. However, the lesion most commonly appears hypointense in T1-weighted images and hyperintense in T2-weighted images, while areas of enhancement within the neoplasm can be demonstrated after gadolinium administration [17,24]. The low signal of the lesion, which is similar to muscles, can be explained in T1-weighted images by the long T1 relaxation time of the neoplastic tissue, which replaces the hyperintense fatty bone marrow. The high signal intensity of the neoplasm in T2-weighted images is caused by its long T2 relaxation time due to the high intracellular and extracellular water content of the neoplastic tissue [24].

Of particular interest is the fact that PBL is characterized by bone marrow changes and surrounding soft tissue mass presence withοut significant cortical destruction [7,24,33]. This finding, best shown by MRI, exclusively concerns small round blue cell tumors, including bone lymphoma, multiple myeloma and Ewing’s sarcoma [3,33]. A possible mechanism explaining the minimal cortical erosion despite the presence of a soft tissue mass was proposed by Hicks et al. [34]. After conducting immunohistochemical studies, the authors suggested that neoplastic cells mediate this process by secreting a subset of osteoclast-stimulating cytokines, namely interleukin (IL) -1, IL-6 and tumor necrosis factor (TNF) -α, causing increased local bone resorption and, consequently, the formation of penetrating channels through the cortex, which allow for the tumor cells to escape the intramedullary space and spread to the surrounding soft tissues without extensive cortical destruction.

The role of ultrasound examination in cases of PBL is limited. It can often depict a soft tissue mass associated with PBL and also demonstrate the typical imaging pattern of bone cortex preservation despite the extraosseous extension of the lesion [21]. Ultrasonography, however, can be helpful in the establishment of a PBL diagnosis via ultrasound-guided biopsy [21,25].

18F-fluorodeoxyglucose (FDG) positron emission tomography (PET) scan is a functional imaging tool capable of detecting viable neoplastic tissue due to its hypermetabolic nature and, thus, its high FDG uptake. PET scans are frequently combined with CT imaging, and recently MRI, allowing for a correlation between functional and anatomical information and, therefore, more accurate localization of metabolic abnormalities [12]. To semiquantify PET findings, lesions are primarily evaluated based on the calculation of their maximum standardized uptake value (SUV_max_) [12,24].

FDG PET/CT is the modality of choice for the exclusion of secondary bone lymphoma and for the staging, restaging, follow-up and treatment response assessment of PBL [24]. If PET/CT indicates bone marrow involvement, a bone marrow biopsy could be omitted unless a discordant histology of the bone marrow would alter treatment. A successful response to treatment manifests as a rapid decrease in FDG uptake compared to the initial imaging and newly depicted FDG-avid lesions can be assumed to be recurrences [12]. An SUV_max_ of 2.5 as a cutoff to distinguish the residual from a metabolically inactive disease achieves a negative prognostic value and sensitivity of 100% to identify residual lymphoma. However, a high false positive rate has been reported, with post-therapeutic osteonecrosis proposed as the main potential cause [35]. In addition, FDG PET/CT can depict soft tissue extension of PBL similar to MRI [24]. The main benefit of FDG PET/CT in staging compared to CT and MRI is its ability to identify previously unknown bone lesions with a whole-body scan [24]. Indeed, Liu et al. [35] and Wang et al. [36] reported that FDG PET/CT revealed additional bone lesions in almost 50% of PBL cases. Overall, PET is highly valuable for the diagnostic process of PBL and is considered to be superior to MRI.

More recently, PET/MRI has become available with encouraging results and offers the opportunity to benefit from the additional information provided by special diagnostic MRI techniques, particularly diffusion-weighted imaging and magnetic resonance spectroscopy [12]. PET/MRI could potentially be the modality of choice for the diagnostic management of PBL patients.

Skeletal scintigraphy is one of the standard imaging tools used to investigate tumors involving the musculoskeletal system [12]. However, it has been shown to present a lower sensitivity and specificity than FDG PET in detecting lymphomatous infiltration of bone [22].

The radiological appearance of PBL can mimic numerous diseases. In young patients, the differential diagnosis of PBL includes metastatic neuroblastoma, Langerhans’ cell histiocytosis, Ewing’s sarcoma, osteomyelitis and leukemia. In older teenagers and young adults, osteosarcoma should also be considered. In older adults, bone metastases and multiple myeloma are much more frequent than PBL. However, a permeative or moth-eaten pattern of destruction and periosteal reaction in the conditions above are not as common as in PBL and, thus, may raise the suspicion of the latter in this age group [33]. Additionally, it is significant to distinguish PBL from secondary bone lymphoma. When sequestrum formation is noted, which is uncommon in PBL, the differential diagnosis must include osteomyelitis, osseous tuberculosis, radiation necrosis and eosinophilic granuloma [24]. Despite its rarity, PBL should be considered a diagnostic possibility in the presence of the aforementioned clinical and radiological findings, particularly due to its different treatment and improved prognosis compared to other primary bone malignancies [33].

In conclusion, the imaging presentation of PBL varies and can be non-specific in some cases [3,15]. However, the demonstration of a solitary, lytic osseous lesion found in the diametaphysis of a long bone with a permeative or moth-eaten pattern of destruction, layered periosteal reaction and minimal cortical disruption in conventional radiography and a soft tissue mass in CT and MRI is highly suggestive of PBL [24,33].

## 5. Histological Findings

The histological and immunohistochemical appearance of PBL has been reported in a number of studies [2,3,4,7,8,9,14,15,16,18,19,20,22,23,24,25,26,27,37,38,39,40,41,42,43,44,45,46,47,48,49,50,51,52,53,54,55,56,57,58,59,60,61,62,63,64,65,66,67,68,69,70,71,72,73,74,75,76,77]. The findings of articles published before 2006 [16,37,38,39,40,41,42,43,44,45,46,47,48,49,50,51,52,53,54,55,56,57,58,59,60,61,62,63,64,65,66,67,68] were summarized in our previous review about PBL [78]. Herein, we analyze the histological and immunohistochemical features of PBL described in subsequent studies [2,3,4,7,8,9,14,15,18,19,20,22,23,24,25,26,27,69,70,71,72,73,74,75,76,77].

The definite diagnosis of PBL is histological with immunohistochemical examination and, therefore, clinical and radiological suspicion of PBL should be further evaluated by histopathology via bone biopsy [2,8,9,22]. Tissue samples can be obtained either via image-guided percutaneous fine-needle, core-needle or open biopsy. Kenan et al. [19] consider core biopsy the preferred method since it ensures an appropriate representative specimen and minimal damage to the surrounding tissue. Collecting a sufficient amount of tissue is of great significance for PBL cases because of the extensive tissue necrosis and damage during cytological preparation that characterize lymphomas and result in reduced diagnostic accuracy [19]. Keeping the amount of resected tissue to a minimum is also important to diminish the risk of pathological fractures in the affected area and, thus, it is recommended to avoid excisional biopsies [22]. However, a unique case of spontaneous resolution of primary bone DLBCL (PB-DLBCL), NOS following incisional biopsy without adjuvant therapies has been reported. This phenomenon occurred via an unknown mechanism, possibly related to the activation of the immune system [27]. In addition, biopsy procedures should be carefully performed to prevent iatrogenic physeal injury [19].

NHL accounts for the vast majority of PBLs with DLBCL, NOS being the predominant histological type, constituting approximately 80% of cases [3,7,8,9,14,19,22,23,24,72,75,76]. Other less frequent pathological NHL types include follicular lymphoma, marginal zone lymphoma, small lymphocytic lymphoma (SLL), lymphoplasmacytic lymphoma, Burkitt lymphoma, B-lymphoblastic lymphoma, anaplastic large cell lymphoma (ALCL), adult T-cell lymphoma/leukemia (ATLL), extranodal natural killer (NK)/T-cell lymphoma and peripheral T-cell lymphoma (PTCL), NOS [3,9,14,19,22,23,24,72,76].

**Figure 1:**
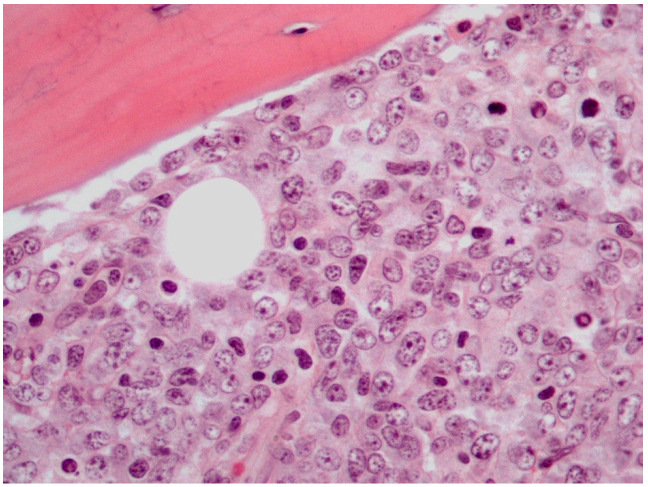
Histological features of PB-DLBCL, NOS (Hematoxylin-Eosin staining, 600× magnification).

The majority of PB-DLBCLs, NOS exhibit a diffuse growth pattern (Figure 1) [8,18,72]. The neoplasm consists of large, atypical cells or a polymorphous mixture of small to large cells with multilobulated nuclei, fine chromatin and an unobtrusive to prominent nucleoli in a background infiltrated by mature T-cells [2,8,18,27,72]. The bone trabeculae can appear normal or thickened. Delicate reticulin fibrosis and occasional diffuse fibrosis are observed between the cells. This lymphoma may uncommonly display immunoblastic morphology, which is characterized by abundant amphophilic cytoplasm and round nuclei with prominent nucleoli. Additionally, PB-DLBCL, NOS may rarely contain clear cytoplasm cells and signet ring cells, thus mimicking metastatic adenocarcinoma [72]. An extremely unusual morphology of PB-DLBCL, NOS is the spindle cell variant, which can be misdiagnosed as sarcoma or carcinoma [23,72]. It has been reported that lymphoma cells may obtain a spindle morphology as they infiltrate into osseous and soft tissues. Furthermore, it has been hypothesized that the lymphoma cells mediate their spindling process by secreting specific cytokines, including TNF-α, platelet-derived growth factor (PDGF) and transforming growth factor (TGF) -β [23]. These cytokines induce fibrosis and proliferation of fibroblasts, which may impinge on neoplastic cells compressing them into a spindle shape [23,72].

**Figure 2:**
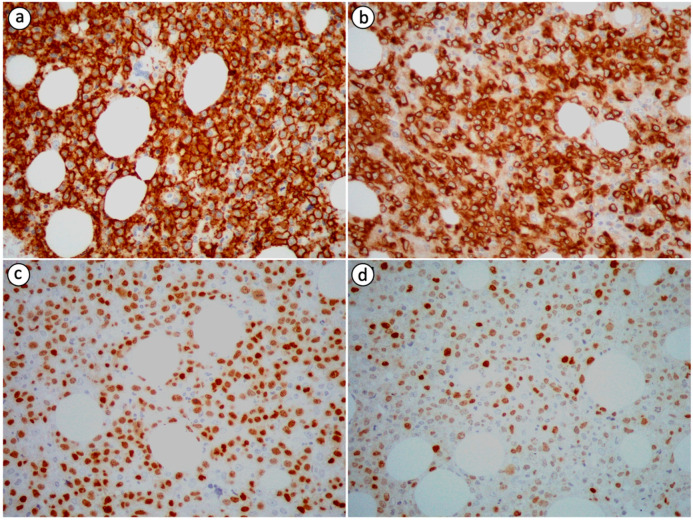
Immunohistochemical staining of PB-DLBCL, NOS (400× magnification) showing positivity of the tumor cells for (**a**) CD20 (most tumor cells are strongly immunopositive), (**b**) CD79a (most tumor cells are strongly immunopositive), (**c**) PAX5 (a substantial proportion of tumor cells are strongly immunopositive) and (**d**) MUM1 (the minority of tumor cells are strongly immunopositive).

Neoplastic cells in PB-DLBCL, NOS are immunohistochemically positive for B-cell markers, including cluster of differentiation (CD) 19, CD20, leukocyte common antigen (LCA/CD45), CD79a and PAX5, and negative for T-cell markers, such as CD3 and CD5, which can, nevertheless, highlight small T-cells in the background [2,8,14,19,20,23,26,27,72]. Immunoreactivity for CD10, B-cell lymphoma 6 protein (BCL-6), multiple myeloma oncogene 1 (MUM1), also known as interferon regulatory factor 4 (IRF4), B-cell lymphoma 2 protein (BCL-2), C-MYC and CD75 is variable (Figure 2) [2,8,15,26,27]. The expression pattern of CD10, BCL-6 and MUM1 can be used to further divide DLBCL, NOS into distinct histogenetic groups using the Hans algorithm [72]. According to the 2016 revised WHO classification for lymphoid neoplasms, the co-expression of C-MYC and BCL-2 without associated rearrangements defines an adverse prognostic marker named double-expressor (DE) lymphoma [8,71]. This diagnostic entity accounts for 20–30% of DLBCL, NOS cases. A case of DE PB-DLBCL, NOS was encountered and reported by Papageorgiou et al. [8]. Primary bone DLBCL, NOS is characterized by a high Ki-67/MIB-1 proliferative index with a mean value of 85% based on the data extracted from the case reports we reviewed [2,8,14,26].

Lima et al. [74] utilized fluorescence in situ hybridization (FISH) to examine 63 cases of PB-DLBCL, NOS and obtained 32 interpretable results. The t(14;18)(q32;q21) translocation was detected in 28% of cases. This percentage is significantly higher than that reported in extranodal DLBCL, NOS cases, but is in accordance with the results found in nodal localizations, suggesting that PB-DLBCL, NOS is closer to nodal than extranodal neoplasms. C-MYC and BCL-2 rearrangements were detected in 9 and 28% of cases, respectively, and one case exhibited rearrangements in both genes, which, albeit rare, is classic in nodal neoplasms. Although BCL-6 is frequently rearranged in DLBCL, NOS of nodal and extranodal localization, Lima et al. [74] reported that BCL-6, as well as anaplastic lymphoma kinase (ALK), PAX5 and B-cell lymphoma 1 protein (BCL-1), also known as cyclin D1, genes were in a germline configuration, i.e., without rearrangements, in all 32 cases they examined. According to the authors, their results suggest that PB-DLBCL, NOS represents a distinct group within the category of extranodal B-cell lymphomas based on their findings. However, subsequent studies reported PB-DLBCL, NOS cases with BCL-6 rearrangements. A clinicopathological study conducted by Bhagavathi et al. [73] showed C-MYC, BCL-2 and BCL-6 rearrangements in 9, 19 and 14% of examined cases, respectively. Furthermore, Li et al. [70] reported rearranged C-MYC, BCL-2 and BCL-6 genes in 29, 25 and 50% of tested cases, respectively, and stated that C-MYC rearrangement frequency was found significantly higher in PB-DLBCL, NOS (29%) compared to non-osseous de novo DLBCL, NOS (12%) and nodal DLBCL, NOS (9%). Future studies might provide new evidence about the significance and clinical relevance of the aforementioned rearrangements.

Hsieh et al. [15] described the microscopic appearance of a primary bone atypical SLL case. Upon morphological inspection, the lesion comprised diffuse sheets of small lymphocytes with scanty cytoplasm and clumped chromatin without Dutcher bodies, plasmacytoid differentiation and mitoses. An immunohistochemistry study revealed positivity for CD20, negativity for CD3, CD5, CD10, CD23, CD43, immunoglobulin (Ig) D, IgM, BCL-6 and BCL-1 and equivocal reactivity for BCL-2. The Ki-67/MIB-1 proliferative index was low with a value of 10%.

PBL of T-cell origin is extremely rare and more common in Japan and Taiwan than in the West [15,16]. Mature T-cell and NK-cell neoplasms represent a category of rare lymphomas and constitute approximately 10–15% of all NHLs [4,69]. This group of lymphomas includes ALCL, which is the most common subtype of T-cell PBL, ATLL and PTCL, NOS, among others [22,69].

ALCL is an uncommon disease, accounting for less than 5% of all NHL cases. Its presentation with isolated osseous involvement is rare [77]. This neoplasm is composed of large-sized cells with pleomorphic nuclei and multiple prominent nucleoli [72,77]. Tumor cells are positive for CD30, also known as Ki-1 antigen, and some of the T-cells markers, such as CD3, CD5 and CD43. Immunoreactivity for CD45, epithelial membrane antigen (EMA), cytotoxic proteins, T-cell intracellular antigen 1 (TIA-1), perforin and granzyme B varies [15,72,77,79]. Most ALCLs carry the t(2;5)(p23;q35) translocation, which results in the fusion of ALK with nucleophosmin (NPM) and can be revealed via immunohistochemistry [22,72,76,77,79]. Immunohistochemical markers, including EMA, cytokeratin, S100, CD1a, CD99, friend leukemia integration 1 protein (FLI1), neuron-specific enolase (NSE) and lymphoid markers, can be used to differentiate ALCL from other neoplasms. Multifocal disease, ALK negativity, necrosis and advanced age are considered adverse prognostic factors of ALCL [72]. However, there is evidence that ALK positivity is not a favorable prognostic marker for primary bone ALCL, unlike nodal ALCL [77].

Jadidi et al. [25] reported an unusual case of primary bone ATLL in a patient infected by human T-cell lymphotropic virus 1 (HTLV-1). The neoplasm consisted of proliferating, atypical, highly pleomorphic lymphocytes with hyperchromatic nuclei in a background of fibrosis and adipocytes. Immunohistochemical staining of the tumor cells showed positivity for CD2, CD3, CD4, CD5, CD25 and CD45 and partial loss of CD7. Immunoreactivity for CD8 was variable among the neoplastic lymphocytes, whereas FOXP3 was expressed in a minor subset of them. The proliferation fraction as determined by Ki-67/MIB-1 immunostaining was high at 60–70%.

PTCL, NOS represents approximately 30–50% of mature T-cell and NK-cell lymphomas [4]. Yu et al. [4] reported a case of PTCL, NOS manifested as PBL. A histopathological examination demonstrated prominently atypical neoplastic cells with various shapes and abundant, pale cytoplasm scattered in a necrotic background. An immunohistochemical study revealed the expression of CD68 and CD163, indicating the presence of M2 macrophages. Another such case described by Hsieh et al. [15] showed lymphoma cells immunohistochemically positive for CD2, CD43, CD45, CD56, TIA-1 and granzyme B and negative for CD3, CD5 and CD30.

Metagenomic next-generation sequencing (mNGS) is a technique that unbiasedly sequences microbial and human nucleic acids from a variety of clinical samples. Therefore, it can detect not only the presence of microorganisms, but also chromosomal copy number variations (CNVs), which can be indicative of a malignancy. Although biopsy and histopathological examination is undoubtedly the gold standard for neoplasm diagnosis, it is an invasive and time-consuming procedure that is not always performed, especially when the presence of a tumor is not primarily considered. mNGS, on the other hand, is a non-invasive and fast technique that can set the suspicion of a malignancy and lead to more focused diagnostic testing for a neoplasm [26]. Indeed, Liu et al. [26] reported a case of recurrent fever and toothache in which peripheral blood mNGS excluded microbial infections and detected CNVs in several chromosomes, suggesting the possibility of a malignancy. A mandible biopsy was conducted and confirmed the presence of primary DLBCL, NOS of the mandible.

## 6. Etiology

Although the etiology of PBL remains unclear, several factors have been incriminated for its development. It has been reported that PBL might be related to other bone disorders (e.g., Paget’s disease and hereditary exostoses), immunological disorders (e.g., sarcoidosis), viral agents, environmental factors and immunosuppression following transplantation or accompanying acquired immunodeficiency syndrome (AIDS) [22,37,80]. Cytogenic and molecular abnormalities, which are known to be involved in the pathophysiology of various types of lymphomas, have been documented in PBL cases as well, such as the t(2;5)(p23;q35) translocation observed in ALCL [37,80,81]. Trauma might also be associated with PBL development, which can be explained by the consequent inflammatory process eventually resulting in local carcinogenesis [18,28,29,30,37]. Lastly, PBL may arise from osteomyelitis most commonly in the setting of immunological diseases or chronic recurrent multifocal osteomyelitis in children, but also in other conditions, such as bacterial and viral infections, particularly Epstein–Barr virus, a history of bone implant, articulation replacements and Paget’s disease of bone [22,80,81,82].

## 7. Histogenesis

DLBCL, NOS has been shown to be morphologically, genetically and clinically heterogenous by numerous studies [69,83,84]. Hence, it can be subclassified into morphological and phenotypic variants and molecular or genetic categories. However, it is recommended that the role of morphological variants, namely centroblastic, immunoblastic and anaplastic, and phenotypic variants, such as CD5-positive, should be de-emphasized due to their weak prognostic implications and the fact that they do not reflect actual biological subgroups [69]. Cell-ff-origin (COO) classification of DLBCL, NOS, on the other hand, is a basic biological subdivision with prognostic impact and should be maintained at this time, although it has been shown that the clinical impact of COO stratification is in fact relatively limited outside clinical trials [69,83]. The histogenesis of DLBCL, NOS has been the research subject of various studies with not only molecular, but also with immunohistochemical criteria.

Gene expression algorithms are used to subdivide DLBCL, NOS into germinal center B-cell-like (GCB), activated B-cell-like (ABC) and an unclassified or type-III group [69]. The GCB subtype is characterized by a gene expression profile (GEP) referring to a germinal center (GC) COO and is enriched for the t(14;18)(q32;q21) translocation, which leads to IgH/BCL-2 fusion, and mutations of genes instrumental for GC development, GC dark and light zone transitions and microenvironmental interactions. In contrast, the ABC subtype derives from GC-exit or post-GC cells with either GC-exit or early plasmablastic phenotype. It is dependent on B-cell receptor (BCR) signaling and NFκB activities, negative for most GC markers, positive for MUM1 and enriched for BCR pathway mutations, as well as genetic changes that block a B-cell differentiation program [83]. It must be mentioned, however, that the results of trials of DLBCL, NOS treatment incorporating targeted agents using COO for patient selection were largely disappointing, which underlines the insufficiency of this binary subdivision and highlights the need for a more molecularly-based approach [69]. Recent studies have independently identified 5–7 new functional DLBCL, NOS subgroups with clinical and prognostic relevance using molecular/cytogenetic profiling, thus supporting the validity of this concept, although they failed to classify all cases [69,85,86,87,88]. Such studies could potentially provide new knowledge about the genetic background of DLBCL, NOS.

Immunohistochemistry algorithms are widely used in routine clinical practice to predict the COO of DLBCL, NOS, although they present concordance issues with gene expression profiling and do not recognize the type-III gene expression category [83]. The Hans algorithm uses the immunohistochemical expression pattern of CD10, BCL-6 and MUM1 to subclassify DLBCL, NOS into GCB and non-GCB groups. CD10 and BCL-6 are markers of GC B-cells, while MUM1 is expressed in later stages of B-cell development and plasma cells. Therefore, cases positive for CD10 or positive for BCL-6 and negative for CD10 and MUM1 are assigned to the GCB group, while the remaining cases belong to the non-GCB group [89]. For example, the DLBCL, NOS case reported by Papageorgiou et al. [8] was immunohistochemically positive for BCL-6 and MUM1 and negative for CD10 and was, hence, classified in the non-GCB group by the authors. Hans et al. [89] reported that their immunohistochemistry algorithm predicted the cDNA classification in 71% of GCB and 88% of ABC or type-III DLBCL, NOS cases.

Interestingly, Dybkær et al. [90] proposed a refined COO classification system for DLBCL, NOS. This system is based on subset-specific B-cell-associated gene signatures and has expanded the COO classification of DLBCL, NOS to incorporate five B-cell subtypes: naïve, centroblast, centrocyte, memory and plasmablast.

Regarding PB-DLBCL, NOS specifically, studies have shown that most cases belong to the GCB subtype [8]. In two large series of PB-DLBCL, NOS cases studied by Li et al. [70] and Lima et al. [74], the percentage of the GCB subtype was approximately 90% and 67%, respectively. Of note, Li et al. [70] found that PB-DLBCL, NOS presented a GCB subtype more frequently than both secondary bone DLBCL, NOS and non-osseous DLBCL, NOS. In a study with a small number of patients, Yousef et al. [11] stratified PB-DLBCL, NOS cases into unifocal and multifocal and found that the GCB subtype was indeed predominant among unifocal cases, but both GCB and non-GCB subtypes appeared equally among multifocal cases.

According to de Groot et al. [91], anatomical localization and age matter with respect to specific COO subtypes of DLBCL, NOS. Studies have shown an apparent correlation between the preferred anatomical localization and the COO subtype. For example, primary bone, primary ovarian and craniofacial DLBCL, NOS and primary mediastinal (thymic) large B-cell lymphoma predominately harbor a GCB subtype, whereas primary breast DLBCL, NOS and primary central nervous system, primary testicular and intravascular large B-cell lymphoma are mainly classified in the ABC subgroup [91]. In addition, GCB PB-DLBCL, NOS predominately constitutes a centrocyte-like profile, while GCB non-osseous DLBCL, NOS mainly presents a centroblast-like phenotype [92]. An association between age and COO subtype has also been observed, with the ABC subtype predominant in the elderly [91].

De Groen et al. [92] studied a total of 166 osseous and GCB non-osseous DLBCL, NOS cases and reported a mainly centrocyte-like phenotype for PB-DLBCL, NOS and a principally centroblast-like phenotype for GCB non-osseous DLBCL, NOS. In addition, PB-DLBCL, NOS exhibited significantly more frequently mutations in GCB-associated genes and superior survival compared to GCB non-osseous DLBCL, NOS. According to the authors, these findings suggest that PB-DLBCL, NOS represents a distinct entity among extranodal DLBCLs, NOS. Furthermore, in a study conducted by Li et al. [70], patients with PB-DLBCL, NOS presented survival rates similar to those with centrocyte-origin GCB non-osseous DLBCL, NOS but significantly superior to those with centroblast-origin GCB non-osseous DLBCL, NOS. GEPs of PB-DLBCL, NOS resembled those of centrocyte-origin GCB non-osseous DLBCL, NOS but differed from GEPs of GCB non-osseous DLBCL, NOS of other COO, namely naïve, memory, plasmablast and especially centroblast, and from GEPs of ABC non-osseous DLBCL, NOS. As demonstrated by miRNA profiling, PB-DLBCL, NOS and centrocyte-origin GCB non-osseous DLBCL, NOS expressed much higher levels of miR-125a-3p, miR-34-3p and miR-155-5p and significantly lower levels of miR-17-5p and miR-17-3p compared to centroblast-origin GCB non-osseous DLBCL, NOS. The authors concluded that their results provide evidence that PB-DLBCL, NOS is clinically distinct and originates from GC centrocytes, which are biologically attributed for its favorable prognosis.

## 8. Treatment and Prognosis

As already mentioned, PBL is a heterogenous and uncommon disease, hindering the conduction of randomized clinical trials. Therefore, there is no standard treatment for this condition and the recommended strategies are derived from retrospective studies. Treatment options include chemotherapy or immunochemotherapy, radiotherapy and surgery [2,10]. Pain management in patients with bone tumors is of great significance. The supportive care for pain should be multimodal with the use of multiple complementary pharmacological and non-pharmacological approaches [93,94].

The role of surgery in the management of PBL is generally limited. Due to the progress of chemotherapy and radiotherapy, most surgical procedures have been performed for diagnostic purposes, namely biopsy [2,4,8,9,10,22]. Indications for surgical treatment of PBL include impeding or actual pathological fractures, neurological complications, such as spinal cord compression, segmental defects in long bones and skeletal or articular collapse caused by avascular necrosis following treatment [2,4,7,8,9,10,24]. Early surgical treatment of lower extremity pathological fractures before chemotherapy provides a better quality of life and helps the patient endure subsequent treatment and hospitalization. In the case of an upper extremity fracture, however, given the minor disability resulting from the use of a brace, surgical treatment can be delayed, allowing for early initiation of chemotherapy and radiotherapy [24]. Orthopedic care is significant during treatment and the recovery period, as the risk for fracture persists until complete bone healing. Patients with involvement of weight-bearing bones may rarely require internal stabilization or bracing until bone healing is achieved [9]. In a study conducted by Yang et al. [10], surgical treatment of PB-DLBCL, NOS did not affect survival.

Treatment of PBL is based on systemic therapy and the current modalities include chemotherapy or immunochemotherapy with or without radiotherapy, resulting in a 5-year overall survival (OS) of approximately 70% [2,7,8,9,10,19,20,21,23]. Anthracycline-based, multiagent chemotherapy comprising cyclophosphamide, doxorubicin, vincristine and prednisone (CHOP) with or without the addition of rituximab (R-CHOP) is the preferred modality [2,9,10,19,20,22,23,24,95]. According to Ramadan et al. [75] the addition of rituximab to CHOP increased the 3-year progression-free survival (PFS) of patients with PBL from 52% to 88%, although Müller et al. [7] reported no difference in the OS between patients treated with CHOP and R-CHOP. Additionally, chemotherapy may decrease the risk of local recurrence in PBL and improve the prognosis of children and adults with disseminated disease [7]. Bruno Ventre et al. [95] reported a favorable prognosis of patients with PB-DLBCL, NOS who underwent chemotherapy with or without radiotherapy and claimed that chemotherapy is more effective than radiotherapy in PB-DLBCL, NOS cases. Indeed, chemotherapy has proven to be superior to radiotherapy for the treatment of PBL with a 10-year OS of 56 and 25%, respectively. However, various studies have shown improved OS with combined chemoradiotherapy compared to chemotherapy or radiotherapy alone [22]. Christie et al. [96] reported a 5-year OS of 90% in PBL patients treated with both chemotherapy with three cycles of CHOP and radiotherapy to a dose of 45 Gy in 25 fractions, whereas Müller et al. [7] reported a trend for better OS with chemotherapy combined with 4-6 cycles of CHOP and radiotherapy to a typical dose of 46 Gy, which was, however, statistically insignificant. Furthermore, Beal et al. [97] found that PBL patients managed with a combined modality versus a single modality therapy presented a significantly superior outcome, with a 5-year OS of 95 and 78%, respectively. Hence, chemotherapy and radiotherapy are usually combined for the treatment of PBL, achieving a 5-year OS of 80–90% [21]. The choice of chemotherapy regimen is based on the histology of PBL. R-CHOP is the preferred regimen when the PBL is of B-cell origin and CHOP when of T-cell origin. When anthracyclines are contraindicated, etoposide or gemcitabine can be used instead.

Radiotherapy was once the standard treatment of PBL with appropriate local disease control, but produced disappointing relapse rates, which led to the introduction of chemotherapy for the management of this neoplasm [9,22,23]. In a study conducted by Ma et al. [98], consolidation radiotherapy significantly improved the prognosis in early stage PB-DLBCL, NOS patients compared to chemotherapy alone, with a 5-year OS of 84.2 and 72.7%, respectively, but not in those with advanced stage disease. The same study reported a significantly increased long-term risk for second primary malignancies in early-stage patients with an age at diagnosis of 18–39 years or appendicular skeleton involvement, but not in advanced stage patients. Furthermore, neither early stage nor advanced stage cases were associated with an increased short-term risk for second primary malignancies. These findings can be partly explained by the fact that early stage, younger age and appendicular site of bone involvement were associated with remarkably extended survival time [98]. Various studies suggest that radiotherapy should only be performed as a consolidation modality [10]. When PBL involves areas associated with significant bone marrow production, such as the pelvis, application of radiotherapy should be carefully considered to avoid hematopoiesis-related complications [9,22].

PBL presents a generally favorable prognosis [8,10,14,19,21,35,70,92,99]. In fact, it is considered to possess the best prognosis of all primary malignant bone tumors and better prognosis than secondary bone lymphoma [4,5]. In a study conducted by Li et al. [70], patients with PB-DLBCL, NOS showed significantly better survival compared to those with secondary bone DLBCL, NOS and non-osseous DLBCL, NOS. The prognosis of PBL in the pediatric population is even better, despite the rapid progression, elevated incidence of micrometastasis and tendency to spread to the central nervous system that characterize PBL in children [14,19]. Suryanarayan et al. [48] studied 31 cases of pediatric PBL and reported a 5-year PFS and OS of 95 and 100%, respectively. In cases of complete response, even elderly people may have a long-term survival [99].

Advanced age is an important, unfavorable predictor of survival in patients with PBL [2,7,10,22,24,75,100,101,102]. Specifically, Demicray et al. [101] reported a 5-year disease-free survival of 90% in patients younger than 60 years and 62% in patients aged 60 years old or older. Furthermore, Jawad et al. [13] found 5-year disease-specific survival rates of 87, 74 and 45% in patients with diagnosis age of less than 30, 30–59 and 60 or more years old, respectively. Other adverse prognostic factors include advanced stage determined by either the Ann Arbor or Lugano classification system, declining performance status, high International Prognostic Index (IPI) score, raised lactate dehydrogenase (LDH) levels, multifocality, soft tissue extension and local relapse, which occurs in approximately 10% of patients [2,5,7,8,19,22,31,75,103,104]. Furthermore, Yang et al. [10] reported a significantly lower survival for tumors in the spine compared to the extremity bones, which may be related to the compression of nerves and the complications of this phenomenon, while Liu et al. [31] found a significant survival disadvantage for axial compared to appendicular or craniofacial lesions. In addition, PBLs of T-cell origin exhibited worse prognosis than their B-cell counterparts in a study conducted by Hsieh et al. [15]. In contrast, complete response to treatment has been identified as a favorable prognostic marker of PBL [102,105].

## 9. Conclusions

PBL is an uncommon malignancy and DLBCL, NOS represents the predominant histological type. Diagnosis of PBL is frequently delayed and requires combined histopathological and immunohistochemical examination. Although CT is the primary examination for radiologically guided biopsy, MRI is the standard method for early detection, while FDG PET/CT is recognized as the modality of choice for the staging, restaging, follow-up and treatment response assessment of PBL and for the exclusion of secondary bone involvement of systemic lymphomas. PBL generally conveys a favorable prognosis, especially when treated with combined chemoradiotherapy. Most PB-DLBCLs, NOS belong to the GCB subtype and specifically originate from GC centrocytes. Primary bone large B-cell lymphoma is currently included among DLBCLs, NOS; however, it could possibly represent a distinct entity within the category of large B-cell lymphomas due to its unique characteristics, namely its specific prognosis, histogenesis, GEP, mutational profile and miRNA signature.

## Data Availability

No new data were created or analyzed in this study. Data sharing is not applicable to this article.
